# Apolipoprotein C1 promotes tumor progression in gastric cancer

**DOI:** 10.32604/or.2023.028124

**Published:** 2023-05-24

**Authors:** QIOU GU, TIAN ZHAN, XIAO GUAN, CHUILIN LAI, NA LU, GUOGUANG WANG, LEI XU, XIANG GAO, JIANPING ZHANG

**Affiliations:** Department of General Surgery, The Second Affiliated Hospital of Nanjing Medical University, Nanjing, 210009, China

**Keywords:** Gastric cancer, Apolipoprotein C1, TME, Immune cell infiltration, Drug sensitivity

## Abstract

**Background:**

Gastric cancer (GC) is a malignancy with the worst prognosis that seriously threatens human health, especially in East Asia. Apolipoprotein C1 (*apoc1*) belongs to the apolipoprotein family. In addition, *apoc1* has been associated with various tumors. However, its role in GC remains unclear.

**Methods:**

Firstly, we quantified its expression in GC and adjacent tumor tissues, using The Cancer Genome Atlas (TCGA). Next, we assessed cell invasion and migration abilities. Finally, we revealed the role of *apoc1* in the tumor microenvironment (TME), immune cell infiltration and drug sensitivity.

**Results:**

Firstly, in TCGA database, it has been shown that elevated expression of *apoc1* was identified in various cancers, including GC, then we found that high expression of *apoc1* was significantly correlated with poor prognosis in GC. Histologically, *apoc1* expression is proportional to grade, cancer stage, and T stage. The experimental results showed that *apoc1* promoted cell invasion and migration. Then GO, KEGG, and GSEA pathway analyses indicated that *apoc1* may be involved in the WNT pathway and immune regulation. Furthermore, we found out the tumor-infiltrating immune cells related to *apoc1* in the tumor microenvironment (TME) using TIMER. Finally, we investigated the correlation between *apoc1* expression and drug sensitivity, PD-1 and CTLA-4 therapy.

**Conclusions:**

These results suggest that *apoc1* participates in the evolution of GC, and may represent a potential target for detection and immunotherapy in GC.

## Introduction

Gastric cancer (GC) threatens human health, especially in Asia. In 2020, GC was ranked fifth among new tumor cases and the leading cause of cancer death [1]. Risk factors for GC include smoking, *Helicobacter pylori* infection, alcohol consumption, high-salt diet, and familial inheritance [2]. Various kinds of radical gastric resection + lymph node dissection, combined with perioperative chemo-radiotherapy are currently commonly used therapies, and perioperative targeted drugs and immune checkpoint inhibitors are also potential therapies [3]. However, the therapeutic effects of GC have not progressed significantly. The median survival of patients with advanced GC is approximately 7–9 months, with a 2-year survival rate of only 10% [4]. Therefore, identifying new diagnostic and therapeutic targets is essential [5].

Apolipoprotein C1 (*apoc1*) belongs to the apolipoprotein family and has the smallest molecular weight among all the apolipoproteins. It participates in several biochemical processes including lipid transport and metabolism [6]. For example, it has been revealed that *apoc1* may induce glomerulosclerosis (GS) by enhancing the cytokine response of macrophages, suggesting that *apoc1* can serve as a promising new therapeutic target in GS [7]. In addition, *apoc1* contributes to the non-invasive diagnosis of mycoplasma pneumonia in adolescents [8]. Gene polymorphisms of *apoc1* are also associated with late-onset Alzheimer’s disease (LOAD) in China [9].

*apoc1* also plays an integral role in tumor metastasis. It is distinctive from other members of the apolipoprotein family as it is a major driver of various aggressive behaviors in tumors, especially immune-regulatory processes, invasiveness, and metastasis. For example, high expression of *apoc1* promotes the invasion and metastasis of breast cancer via the Epithelial Mesenchymal Transition (EMT) and JNK/MAPK pathways [10]. *apoc1* is also upregulated in T4 and T3 compared with T2 in colorectal cancer (CRC) and is highly associated with immune cell infiltration, indicating that it can serve as an indicator of metastasis in CRC [11]. Similarly, *apoc1* promotes renal cell carcinoma (RCC) proliferation, invasion, and metastasis by directly targeting Wnt family member 3A (*wnt3a*) [12]. In hepatocellular carcinoma (HCC), single-cell sequencing results have shown that *apoc1* promotes the transformation of M2 macrophages into M1 macrophages, thus promoting the metastasis of HCC, reshaping the tumor immune microenvironment, and improving the immunotherapy effect of anti-PD-1 against HCC [13]. In GC, *znf460* promotes *apoc1* transcription by binding to its promoter, thus promoting GC metastasis through the EMT pathway. Thus, it can serve as a novel biomarker [14,15]. However, its role and specific mechanisms in GC progression remain unclear.

## Materials and Methods

### Gene profile and clinical information of GC and adjacent-tumor tissues

We downloaded data we need from The Cancer Genome Atlas (TCGA) database (https://portal.gdc.cancer.gov), which includes 343 GC and 30 tumor-adjacent tissues. Expression data were converted using the R software (version 4.2.1) for subsequent analysis.

### Analysis between clinical information and apoc1

After downloading clinical information, we evaluated the relationship between them and *apoc1*. Clinical information included age, sex, grade, and stage, T, N, M, and *apoc1*. After comparison, a relevant heat map was drawn based on previous results. “Limma” and “ggpubr” packages were included.

### Cell obtain and culture

GC cell lines (AGS, MKN45) were purchased from the Type Culture Collection of the Chinese Academy of Science (Shanghai, China) and fed MKN45 into RPMI 1640 (Servicebio, Wuhan, China). AGS cells were cultured in F12K (Servicebio, Wuhan, China). All media were supplemented with fetal bovine serum (FBS) (Gibco, NY, USA) and a penicillin-streptomycin mixture. The cells were cultured in incubator with 5% CO_2_ at 37°C.

### IHC assay

*apoc1* protein expression was detected in GC and adjacent tissues by IHC. Formalin-fixed paraffin-embedded tissues sections were degreased in xylene and rehydrated in different concentrations of alcohol and distilled water before antigen retrieval, then followed by antigen retrieval. The fixed sections were washed three times in phosphate buffered saline (PBS) (pH 7.3), incubated in 3% H_2_O_2_ for 7 min, and then washed three times with PBS again. The sections were blocked with BSA (Servicebio, Wuhan, China) for 30 min, followed by incubation with Anti-*apoc1* primary antibody (Abcam, USA) at 4°C overnight, followed by incubation with anti-rabbit antibodies (Proteintech Technology, Wuhan, China) for 50 min. Subsequently, the sections were incubated with diaminobenzidine as a chromogen for 5 min. The sections were then counterstained with hematoxylin and dehydrated using ethanol and xylene. The sections were viewed under an inverted microscope (Nikon Corporation, Japan).

### RT-qPCR

Total RNA was extracted from the GC cell lines using TRIzol regaent (Invitrogen, Carlsbad, USA). Hiscript III Reverse Transcriptase (Vazyme, Nanjing, China) was used for cDNA synthesis. cDNA was quantified using the ChamQ SYBR Color qPCR Master Mix (Vazyme, Nanjing, China) in StepOnePlus Real-Time PCR System (Applied Biosystems, Carlsbad, USA). Relative expression of *apoc1* was calculated using the 2^−ΔΔCT^ method. Primers used are listed in Table 1.

**Table 1:** Part of primer sequences used for qRT-PCR

Gene	Forward	Reverse
ACTB	5′-AGCGAGCATCCCCCAAAGTT-3′	5′-GGGCACGAAGGCTCATCATT-3′
APOC1	5′-GAAGGAGTTTGGAAACACACTG-3′	5′-CATCTTGGCAGAAAGTTCACTC-3′

### RNA transfection

Small interfering RNA targeting *apoc1* and a negative control were acquired from RiboBio (Guangzhou, China). Cells were cultured overnight in serum-free medium before transfection. The cells were then transfected with si-RNAs or si-NC using Lipofectamine 3000 (Invitrogen, Carlsbad, USA).

### Western blot

Total cell proteins were isolated in NP-40 buffer (Beyotime, Shanghai, China), and proteins were separated via SDS-PAGE, followed by transfer onto PVDF membranes (Invitrogen, Carlsbad, USA). The membranes were then blocked with BSA solution for 2 h. Finally, the membranes were incubated with the corresponding primary antibodies at 4°C overnight. After washing with TBST, the membranes were incubated with the corresponding secondary antibodies. The antibodies used in this assay were Anti-*apoc1* (Abcam, Cambridge, UK) and Anti-*gapdh* (Proteintech Technology, Wuhan, China).

### Cell migration and invasion assay

Cells were cultured in serum-free medium and seeded into the top of Transwell chambers (Corning, Shanghai Shang, China) with or without Matrigel-coated filters (BD, NY, USA). Each chamber contained 2 × 10^4^ cells per well. A medium containing 10% FBS was added to the bottom of each chamber. 24 h after incubation, cells were stained with 1% crystal violet. Finally we counted the cell number using the ImageJ software.

### Wound healing assay

First, cells were seeded into 6-well plates. When the density of cells reached at least 80% after transfection, a scratch was made in the middle of the well using a yellow pipette tip. The tip was maintained vertically at the bottom of the well. PBS was then adopted to wash the plate three times. The same scratch field was photographed at the indicated time (0, 24 and 48 h).

### Animal experiments

The animal work was approved by Nanjing Medical University. For the metastasis model, cells transfected with si-RNA or si-NC were injected into the caudal vein of anesthetized nude mice (three mice per group). 14 days later, the lungs of nude mice were removed to count metastatic foci.

### Identification of relevant genes

We analyzed gene expression profile of GC patients using the ggpubr package, and relevant genes were defined with *p* < 0.05, along with corFilter ≥0.2, or ≤−0.2. After screening, scatter plots were drawn based on our results using the “ggExtra” package.

### Identification of differential expression genes (DEGs)

We divided the patients with GC into two groups based on the mean value of *apoc1*. DEGs were defined as *p* < 0.05, and logFC ≥1, or ≤−1. Specific genes are shown in the heat map. Limma and pheatmap packages were included.

### Functional enrichment analysis of DEGs

The “clusterProfiler” package was used for functional enrichment analysis. The threshold conditions were as follows: *p* < 0.05, *q* < 0.2.

### TME and immune cell infiltration

We used the “estimate” package to calculate the TME scores of the GC samples. The TME score consists of two parts: stromal and immune. We then compared these differences based on the expression of *apoc1*. Finally, the “reshape2” and “ggpubr” packages were adopted for the violin plot.

To determine the relationship between *apoc1* and immune cell infiltration, we first used CIBERSORT (http://cibersort.stanford.edu/) to calculate the percentage of immune cells. Next, the “reshape2” and “ggpubr” packages were also used in our study for box plot and scatter diagrams. Then, we drew the correlation circle diagram according to our previous results. Finally, we acquired a list of immune checkpoints and their expression profile data, and screened out checkpoints that are associated with *apoc1*.

### Drug sensitivity and immunotherapy analysis

To calculate the sensitivity of the targeted drugs, we used the “pRRophetic” package. Then, *apoc1* was analyzed and combined with the IC50 results to draw a boxplot. Similarly, we downloaded the immunotherapy scores from the TCIA website (https://tcia.at/) and analyzed the immunotherapy score and *apoc1* expression.

### Statistical analysis

Data are presented as the mean ± standard deviation, and analysis was conducted using GraphPad Prism 8.0 software (GraphPad Software Inc., La Jolla, CA, USA) and ImageJ (National Institutes of Health, USA). We also applied Student’s *t*-test for comparing the experimental groups and control groups. Statistical significance was set at *p* < 0.05.

## Results

### Apoc1 is elevated in GC, indicating a poor prognosis

**Figure 1:**
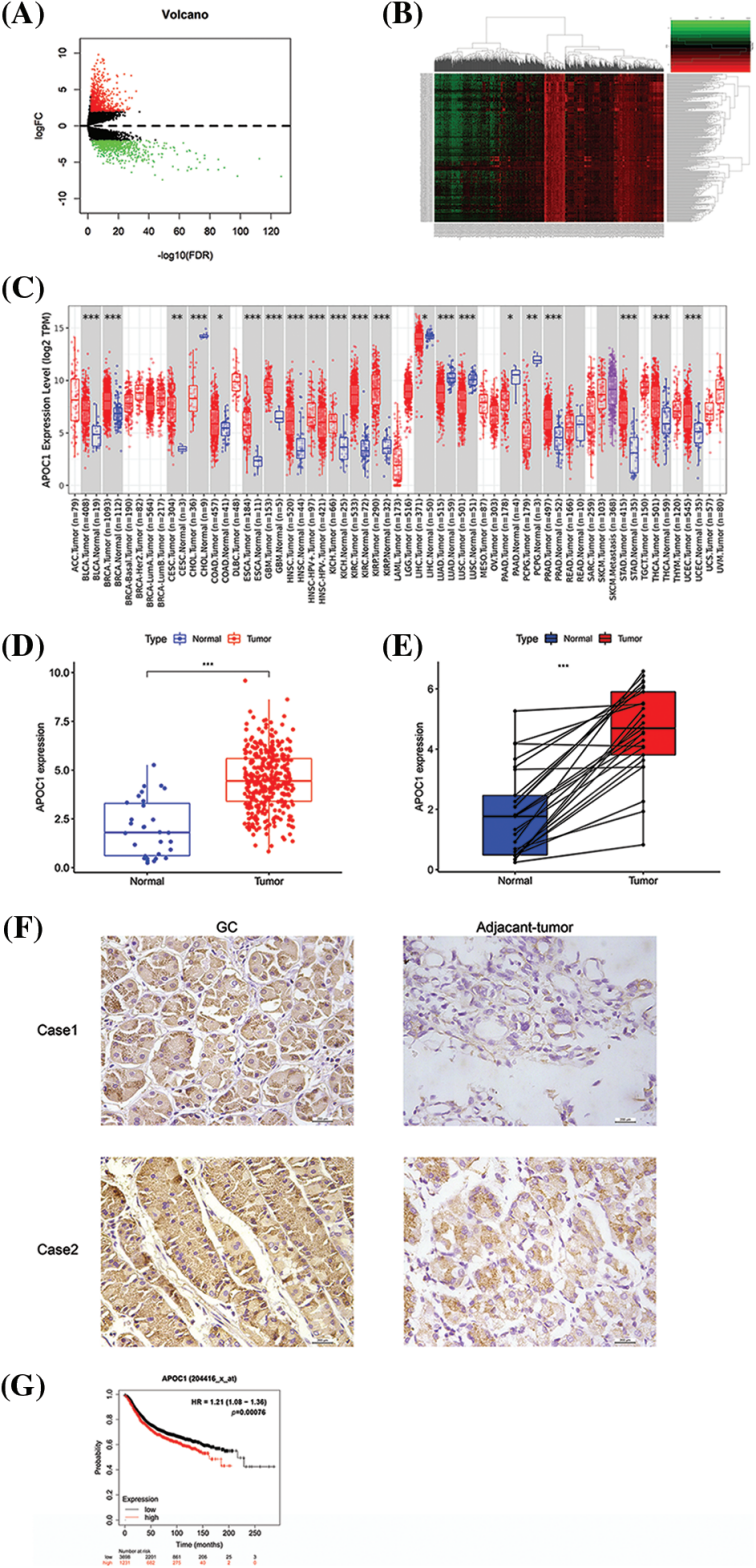
*Apoc1* is elevated in GC, indicating a poor prognosis (A–B) Heat map and volcano map we adopted after differential analysis of TCGA GC data (C) Data of apoc1 expression in various kinds of cancers, including GC (D–E) Data of apoc1 expression levels in GC and adjacent-tumor tissues in TCGA dataset (F) Expression patterns in GC and para-carcinoma tissues revealed by immunohistochemistry analysis. (G) Survival analysis of apoc1 in GC patients TCGA, The Cancer Genome Atlas; GC, gastric cancer; *p < 0.05; **p < 0.01; ***p < 0.001.

First, we conducted a differential analysis of the data of GC patients from the TCGA database, and filtered out 1621 differential genes, with |logFC| ≥ 2 and *p* < 0.05. These differential genes included 872 highly expressed genes and 749 genes with low expression. Based on our results, we drew volcano and heat maps (Figs. 1A and 1B). Among these differentially expressed genes, we chose *apoc1* as the target of our study. We obtained its expression profile in 33 human tumors from the TIMER database (http://TIMER2.0 (cistrome.org)) and found that *apoc1* is abnormally expressed in various types of human tumors, including GC, suggesting that *apoc1* is a tumor-related gene (Fig. 1C). We compared the *apoc1* expression profile between GC and adjacent tumor tissues and found that *apoc1* was significantly elevated in GC (Figs. 1D and 1E). Immuno-histochemical studies also demonstrated significant upregulation of *apoc1* in GC tissues compared with adjacent tissues (Fig. 1F). Finally, we conducted survival analysis based on survival time and the *apoc1* expression profile from the KM plot database (http://kmplot.com/analysis/), and the results indicated that *apoc1* expression was inversely proportional to patient prognosis (Fig. 1G). Overall, *apoc1* expression was elevated in GC, indicating a poor prognosis.

### Apoc1 is associated with tumor stage in GC

**Figure 2:**
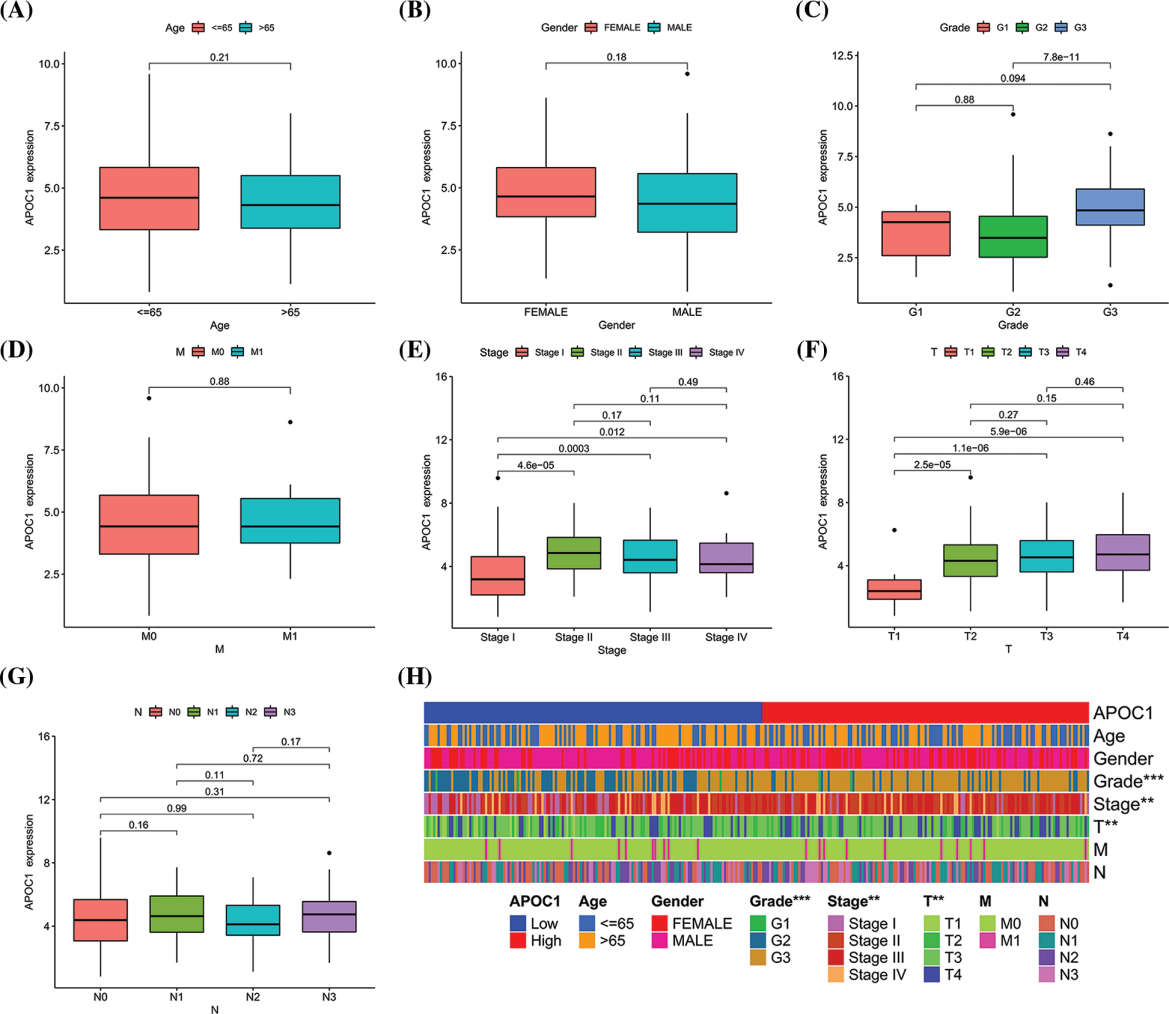
*Apoc1* is associated with tumor in GC (A) age (B) gender (C) grade (D) M (E) stage (F) T and (G) N in GC patients from TCGA (H) clinic-pathological factors relevant heat map. **p < 0.01; ***p < 0.001.

We first divided the samples into different groups based on their clinical and pathological features and compared the expression of *apoc1*. No statistical differences were observed in age (Fig. 2A), sex (Fig. 2B), grade (Fig. 2C), M (Fig. 2D) and N (Fig. 2G) in *apoc1* expression. Compared with the T1 stage, *apoc1* expression was significantly increased in the T2, T3 and T4 stages (Fig. 2F), and the same phenomenon was observed in Stages I, II, III, and IV (Fig. 2E). Finally, we created clinically relevant heat maps to confirm our conclusions (Fig. 2H).

### Down-regulation of apoc1 inhibits abilities of migration and invasion

**Figure 3:**
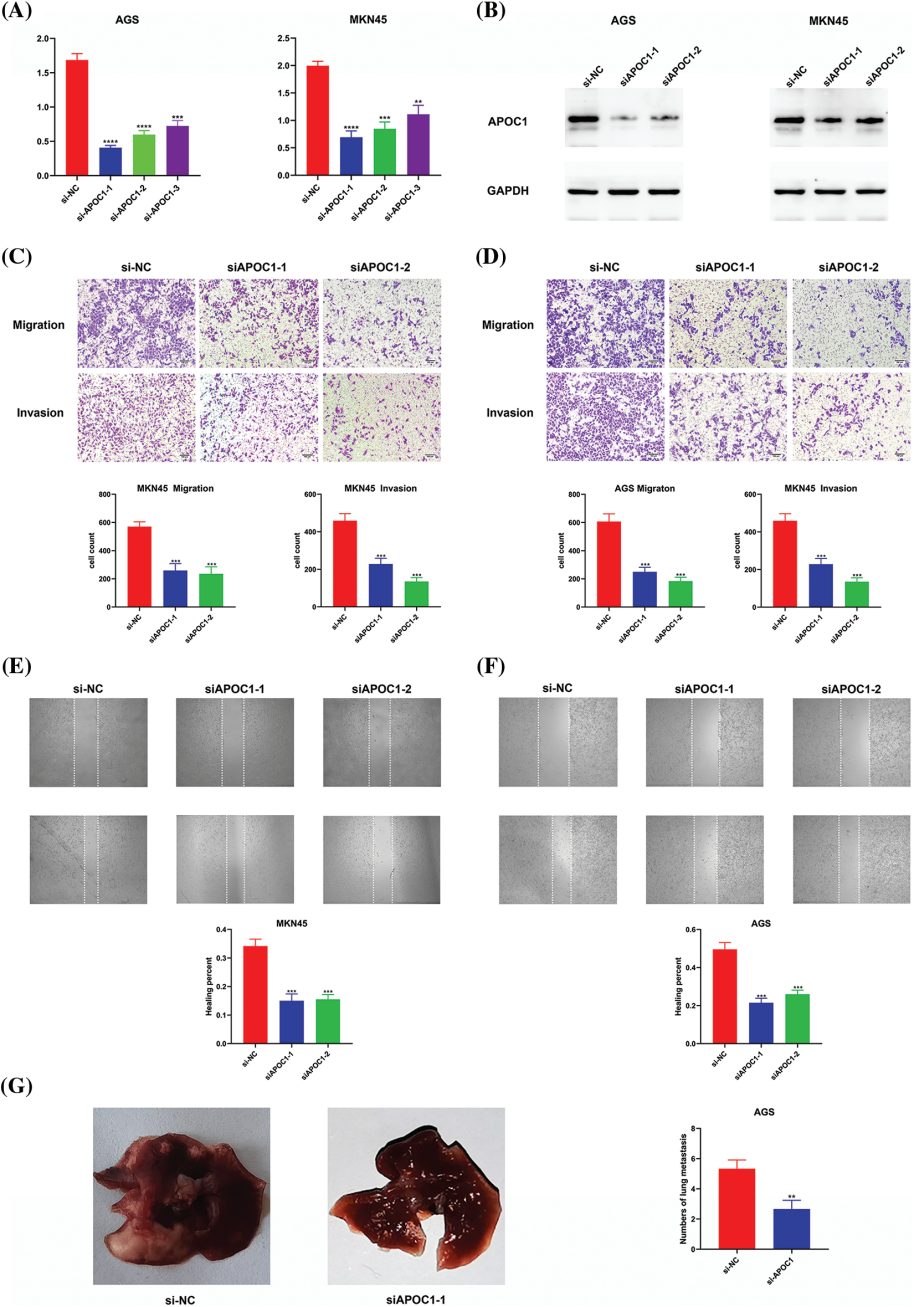
Down-regulation of *apoc1* inhibits abilities of migration and invasion. (A) Efficiency of si-RNAs targeting *apoc1* confirmed by qRT-PCR (B) Efficiency of si-RNAs targeting *apoc1* confirmed by Western Blot (C–D) Transwell assay and (E–F) Wound healing assay were performed undergone different treatments (G) Model of pulmonary metastasis from nude mice **p < 0.01; ***p < 0.001; ****p < 0.0001.

We obtained three siRNAs targeting *apoc1* and a negative control for *apoc1* knockdown. To confirm this efficiency, we performed qRT-PCR and western blotting (Figs. 3A and 3B). We then conducted cell migration and cell invasion assays, and the results showed that the previously mentioned abilities of GC cells transfected with siRNAs were reduced (Figs. 3C and 3D). Furthermore, this was confirmed by the results of the wound healing assay (Figs. 3E and 3F) and the model of pulmonary metastasis (Fig. 3G). Thus, we conclude that *apoc1* plays a major role in cell migration and invasion.

### Apoc1 may be involved in the Wnt pathway and immune regulation

**Figure 4:**
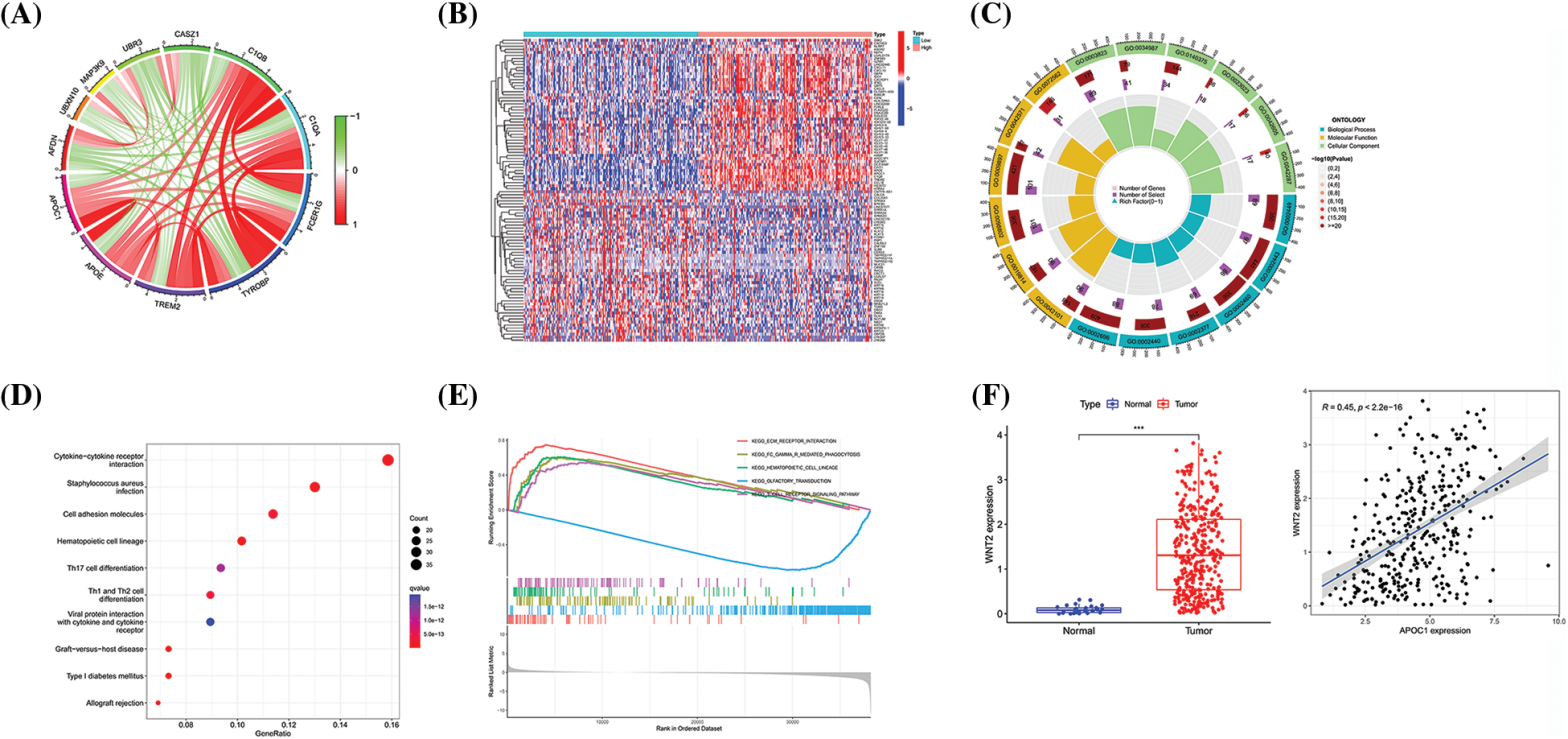
*Apoc1* may be involved in the Wnt pathway and immune regulation (A) Circle diagram of genes which are strongly related to *apoc1* (B) Heat map of differential genes (C) GO analysis (D) KEGG analysis and (E) GSEA analysis of differential genes *apoc1* (F) Expression patterns of *wnt2* in GC and Pearson coefficient between *apoc1* and *wnt2* ***p < 0.001.

To explore the mechanism by which *apoc1* regulates the progression of GC, we conducted analysis to investigate the correlation between *apoc1* and other genes in TCGA database. With |cor| ≥ 0.2 and *p* < 0.05, a total of 2752 correlated genes were identified and correlation circle maps were drawn (Fig. 4A).

Next, we analyzed and adopted 816 differentially expressed genes (DEGs) based on *apoc1* expression, with |logFC| ≥ 1 and *p* < 0.05, as the standard and drew a heat map (Fig. 4B). GO, KEGG, and GSEA analyses of the DEGs showed that *apoc1* was closely related to the Wnt pathway and immune regulation (Figs. 4C and 4E). We found that *apoc1* may participate in Wnt signaling via *wnt2* (Fig. 4F). Overall, *apoc1* may participate in GC progression via the Wnt axis and immune activities.

### Apoc1 relates to TME and immune cell infiltration

**Figure 5:**
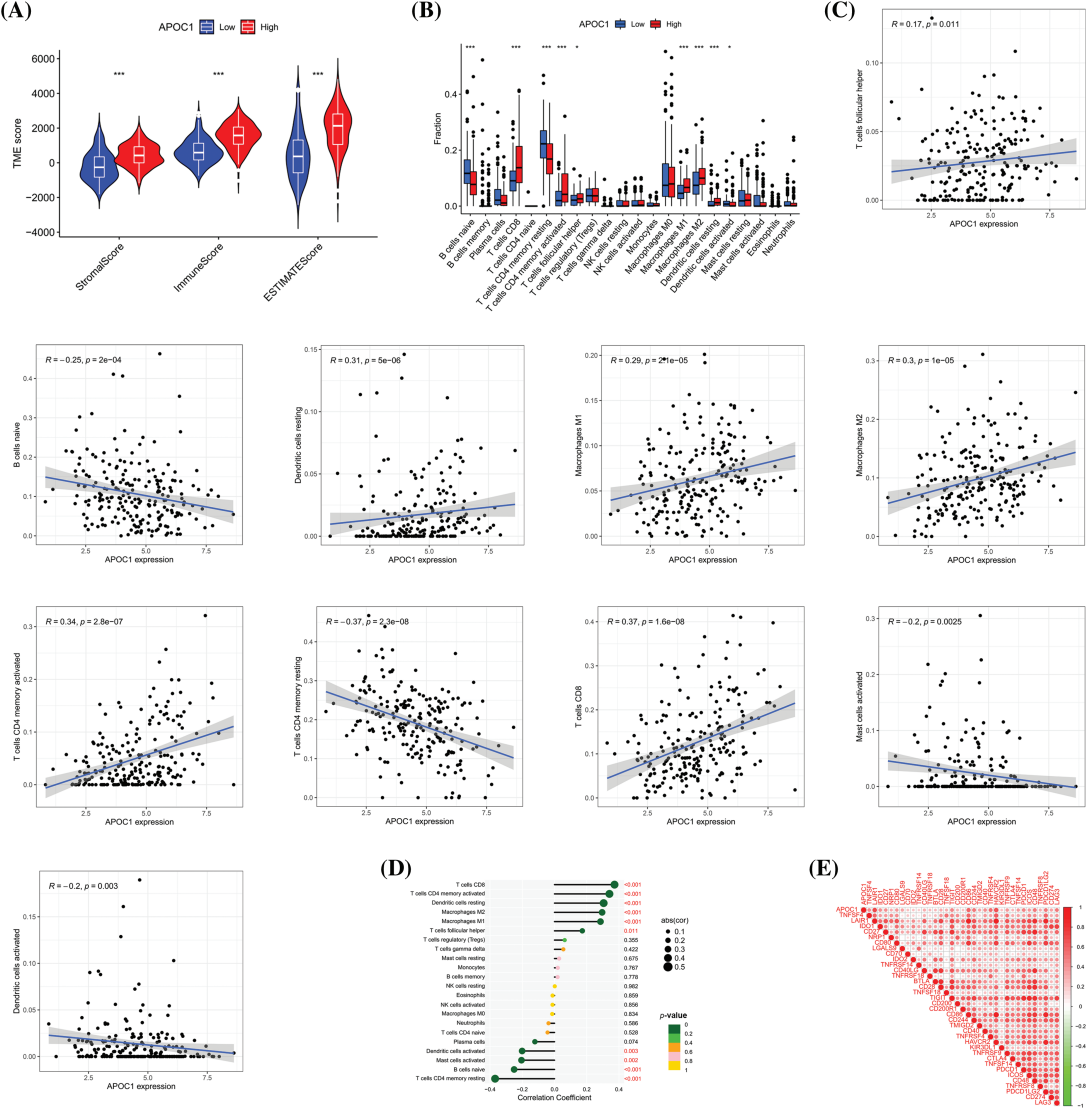
*Apoc1* relates to TME and immune cell infiltration (A) Analysis of differences in TME scores (B) Analysis of differences in immune cells (C) Scatter plot of relevant immune cells (D) Correlation analysis of immune cells and (E) Immune checkpoint *p < 0.05, **p < 0.01, ***p < 0.001.

We further investigated its role in the TME. The results revealed that the stromal and immune scores of the apoc1 high expression group were higher (Fig. 5A).

We then studied the relationship between the fraction of immune cells and the expression profile of *apoc1* and found that its expression was related to the proportion of various immune cells (Fig. 5B). Next, we analyzed the correlation between the proportion of these immune cells and *apoc1*. The proportion of CD8^+^T cells, memory activated CD4^+^T cells, resting dendritic cells, Macrophages M1 and Macrophages M2 were positively proportional to *apoc1*, while the proportion of activated dendritic cells, activated mast cells, naïve B cells, and resting memory CD4^+^T cells were negatively proportional to their expression (Fig. 5C). Subsequent analysis of the immune cell correlation confirmed our conclusion (Fig. 5D). Finally, we obtained immune checkpoints related to *apoc1* (Fig. 5E).

### Drug sensitivity and immunotherapy analysis of apoc1

**Figure 6:**
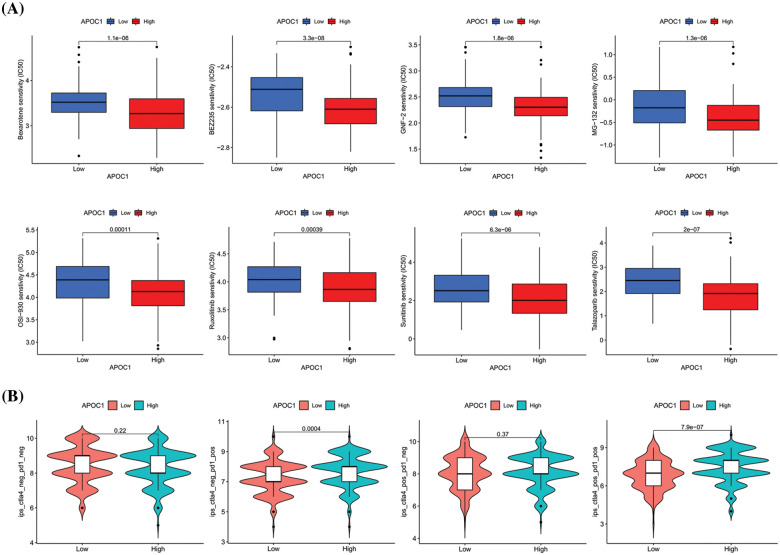
Drug sensitivity analysis of *apoc1* (A) various kinds of small molecule drug whose IC50 are associated with APOC1 expression (B) Relationship between PD-1 and CTLA-4 effects and APOC1 expression.

Targeted immunotherapy is a widely used treatment for advanced GC. To determine the relationship between *apoc1* expression and the sensitivity of targeted drugs, we analyzed the expression and IC_50_ of various targeted drugs in TCGA GC samples, and found that the sensitivity of GC patients to Bexarotene, BEZ235, GNF-2, MG-132, OSI-930, Ruxolitinib, Sunitinib and Talazoparib was relevant to the expression of *apoc1*, and the IC_50_ of these targeted drugs in the *apoc1*-high group was lower (Fig. 6A), suggesting that doctors can decide which one to utilize based on the level of *apoc1* in GC patients.

Among the various immunotherapy regimens, PD-1 and CTLA-4 are the most commonly adopted. To study whether the expression of *apoc1* affects the curative effect of PD-1 and CTLA-4, *apoc1* expression and immunotherapy scores in GC patients were analyzed. The results indicated that the immune scores of the *apoc1* highly group were higher than those in the low group, regardless of whether CTLA-4 was positive or not (Fig. 6B). However, whether CTLA-4 was positive or not and whether its curative effect was related to the expression of *apoc1* was not significantly correlated with *apoc1* expression. In summary, we found some targeted drugs that may have better efficacy in the *apoc1* overexpression group, and *apoc1* may play a major role in PD-1 therapy.

## Discussion

Apolipoprotein is a crucial structural component of lipoproteins and has been shown to be involved in their synthesis, secretion, and catabolism [16]. So far, eight human apolipoproteins (*apoca1, apoca2, apoca4, apocb, apoc1, apoc2, apoc3*, and *apoce*) have been identified. Apolipoprotein is associated with genetic susceptibility to atherosclerosis in humans, and investigation of the regulatory mechanism of apolipoprotein expression is helpful in developing new diagnostic and treatment tools for atherosclerosis [17]. For example, variation in Apolipoprotein E is one of the genetic factors that affect plasma lipoproteins and can be used to diagnose patients with type III hyperlipidemia and screen susceptible populations [18]. Similarly, *apoa4* glycosylation (*G-apoa4*) is associated with coronary artery disease (CAD) in patients with type 2 diabetes mellitus (T2DM), and can induce atherosclerosis via *nr4a3* [19]. *apob* is a key structural protein of atherosclerotic lipoproteins, and plasma *apob* concentration can be used as a measure of the amount of atherosclerotic lipoproteins [20]. *apoc3* is a small protein that regulates TG metabolism and promotes atherosclerosis in humans by interfering with lipoprotein function and catabolism [21].

With further study of apolipoproteins, it was found that apolipoprotein family members also play significant roles in other physiological and biochemical processes, including different diseases [22]. For example, hereditary *apoa1* amyloidosis, which is caused by pathogenic mutations in *apoa1*, can result in amyloid protein deposition in the heart, kidneys, nerves, larynx, and skin [23]. Another study has shown that low level of HDL-C and *apoa1* indicates poor prognosis and recurrence in autoimmune encephalitis (AE): therefore, they may be utilized as biomarkers in the future [24]. An elevated *apob/apoa1* ratio can be used to screen for metabolic syndrome (Met) and insulin resistance (IR) in patients with polycystic ovarian syndrome (PCOS) [25]. Decreased plasma levels of *apob* in patients with non-alcoholic fatty liver disease (NAFLD) impairs the anti-inflammatory ability, and the impaired anti-inflammatory activity is independently correlated with NAFLD [26]. Another study showed that high *apob* expression is associated with an increased incidence of CKD and metabolic dysfunction [27]. Fragments of *apoe* co-localize with neurofibrillary tangles and amyloid β (Aβ) plaques, which may lead to neurodegenerative changes that lead to Alzheimer’s disease (AD) [28].

Members of the apolipoprotein family have also been observed in human malignant tumors [29]. For instance, one study showed that the up-regulation of *apoa1* in cervical squamous cell carcinoma cell lines can promote carboplatin resistance [30]. Plasma levels of HDL-C and *apoa1* are up-regulated in non-small cell lung cancer patients with the epidermal growth factor receptor (EGFR) T790M mutation (NSCLC), suggesting that they have potential as markers in these patients [31]. In a retrospective analysis of *apob*, the preoperative *apob/apoa1* ratio was used to diagnose osteosarcoma in juveniles [32]. In clear cell renal cell carcinoma (ccRCC), preoperative *apob* can be utilized to predict prognosis of patients [33]. In GC, *apoc2* is elevated in peritoneal metastasis (PM) tissues and promotes PM via *cd36*-mediated PI3K/AKT/mTOR signaling [34]. One study suggested that *apoe* is elevated in patients with NPC and promotes tumor growth, migration, and invasion, which may serve as a potential biomarker for the diagnosis of NPC [35].

*Apoc1* is an inhibitor of LDL and VLDL receptors and it can also inhibit the cholesterol ester transfer protein and uptake of fatty acids [36]. Four crystal structures of *apoc1* have been identified, and these suggest a number of physiological functions [37], such as immunity, sepsis, diabetes, cancer, and viral infectivity. For example, it has been suggested that *apoc1* may be used for diagnosis and prognosis of abdominal aortic aneurysm (AAA) [38]. Another study revealed that *apoc1* can help detect metabolic abnormalities early in women with PCOS [39]. In sepsis, *apoc1* may play a protective role against infection by regulating the response to LPS [40].

*Apoc1* is also involved in tumor progression. In colorectal cancer (CRC), *ZEB1-AS1* promotes liver metastasis via the *miR-335-5p*/*APOC1* axis [41]. APOC1 expression is also upregulated in ccRCC and is significantly associated with clinico-pathological factors. *apoc1* participates in cell growth and metastasis in ccRCC [42,43]. Knockdown of *apoc1* also inhibits cell proliferation and induces apoptosis in pancreatic cancer [44].

Programmed cell death protein 1 (PD-1) is a well-known immune checkpoint receptor secreted by activated T cells. Antibodies, including PD-1 and PD-L1, are new drugs for tumor immunotherapy. Although it cannot kill tumor cells directly, it exerts anti-tumor effect by enhancing the patient’s own immune system [45]. PD-1/PD-L1 inhibitors may help to improve the prognosis of triple-negative breast cancer (TNBC) [46]. Similarly, another study suggested that approximately one-fifth of patients with anal squamous cell carcinoma (SCCA) benefit from PD-1 inhibitors [47]. Another meta-analysis showed that PD-1/PD-L1 inhibitors significantly improved the therapeutic efficacy of lung cancer patients with brain metastases compared to chemotherapy [48]. Anti PD-1 combined with SABR for metastatic NSCLC leads to a high response rate and prolongs the clinical benefits of immunotherapy and new systemic therapy by delaying further progression [49].

Antibodies against PD-1 and PD-L1 produce long-lasting responses in a large number of tumor patients; however, most of them eventually relapse due to acquired resistance [50]. For example, up-regulation of *circhmgb2* in LUAD and LUSC can accelerate the progression of LUAD and LUSC by regulating the TME, indicating a new therapy for LUAD and LUSC [51]. *ripk2* has also been reported to induce immunotherapy resistance by triggering cytotoxic T-lymphocyte dysfunction [52]. Multiple clinical trials have shown that LAG-3 blockade alleviates resistance to PD-1 inhibitors in pancreatic cancer. Therefore, the synchronous inhibition of LAG-3 and PD-1 may be a new strategy for alleviating tumor immunotherapy resistance [53]. PD-1 is a promising antitumor therapy; therefore, it is imperative to further study and eliminate acquired drug resistance in patients.

## Data Availability

The datasets used and/or analyzed during the current study will be made available from the corresponding author on reasonable requests.
